# Lexical access failure in Alzheimer’s Disease through the lens of semantic knowledge

**DOI:** 10.1002/alz.085051

**Published:** 2025-01-03

**Authors:** Anamika Banerjee, Azizuddin Khan

**Affiliations:** ^1^ Psychophysiology Laboratory, Indian Institute of Technology, Bombay, Mumbai, Maharashtra India

## Abstract

**Background:**

Lexical access refers to the generation or retrieval of conceptual representation. Representation of concepts or semantic knowledge involves the organization of concepts in an associative manner building up the mental lexicon. Several studies have pointed out an early presence of lexical access failure in Alzheimer’s Disease(AD). While lexical access deficit is rampant among patients with AD, the underlying processes involved in retrieval failure remain debatable. Some findings suggest that the difficulty in lexical access can be due to a deterioration in the conceptual representation. Therefore, to understand the nature of the difficulty underlying lexical access failure among people with AD, the current study aims to understand the involvement of semantic knowledge in lexical access.

**Method:**

In this study, three groups of participants will be recruited, Healthy Controls (HC), Mild Cognitive Impairment (MCI) and Dementia with AD. According to apriori sample size calculation, a total sample size of 54 would be required for the present study, with 18 participants per group. In the current study, the semantic priming task is used to understand the ongoing debate about the involvement of semantic knowledge in Lexical access. The experiment consists of 4 tasks to probe their semantic knowledge and visual perception. Each trial consists of a prime‐ image or word, presented for some duration followed by a related or unrelated target. The participant needs to report whether the target was related to the prime or not.

**Result:**

The lexical retrieval is expected to be differentiated along the different phases of the disease as a function of semantic priming. It is expected that the lexical retrieval in the HC group would be least impacted across the three conditions and AD with Dementia to be the most impacted. The performance on each condition could be differentiated across each disease severity group, along with a difference in the prime type, that is, primes related to the target would show better performance than unrelated primes across all the conditions in each of the groups.

**Conclusion:**

The findings from this study would contribute towards understanding processes underlying lexical access failure among people with Alzheimer’s Disease.

